# Detecting SARS-CoV-2 Omicron B.1.1.529 Variant in Wastewater Samples by Using Nanopore Sequencing

**DOI:** 10.3201/eid2806.220194

**Published:** 2022-06

**Authors:** Lasse D. Rasmussen, Stine R. Richter, Sofie E. Midgley, Kristina T. Franck

**Affiliations:** Author affiliation: Statens Serum Institut, Copenhagen, Denmark

**Keywords:** COVID-19, SARS-CoV-2, severe acute respiratory syndrome coronavirus 2, coronaviruses, viruses, respiratory infections, variants of concern, wastewater surveillance, nanopore sequencing, emerging variants, zoonoses, Denmark. Omicron, B.1.1.529

## Abstract

We report wastewater surveillance for SARS-CoV-2 variants of concern by using mutation-specific, real-time PCR and rapid nanopore sequencing. This surveillance might be useful for an early warning in a scenario in which a new variant is emerging, even in areas that have low virus incidences.

To limit spread of novel SARS-CoV-2 variants such as Omicron B.1.1.529, early detection is crucial. Wastewater surveillance has been suggested as an early warning system for SARS-CoV-2 spread in low-prevalence areas or communities where human testing is limited (*1*).

We provide a method to rapidly determine the presence of Omicron in wastewater samples that have low viral load, in which the Omicron genome represents a minor fraction of the total SARS-CoV-2 genomes. Unlike previously published methods relying on time-consuming, full-genome sequencing and complex variant analysis (*2*), we used a metagenomics approach of long reads containing all differentiating mutations.

For the wastewater surveillance system in Denmark, 24-hour composite samples are collected 3 times/week at the inlet of wastewater treatment plants (WWTPs) throughout the country. Initial RNA purification and real-time quantitative reverse transcription PCR (RT-PCR) analysis is performed by a commercial laboratory (Eurofins Environment Testing Denmark, https://www.eurofins.com), and RNA from SARS-CoV-2‒positive wastewater samples is sent to Statens Serum Institut (SSI) for variant analysis.

On November 26, 2021, the Word Health Organization declared Omicron to be a variant of concern (*3*). On November 29, the commercial laboratory initiated prescreening of wastewater samples for Omicron by using a real-time quantitative RT-PCR targeting the K417N amino acid substitution. Based on the mutation scheme reported by the World Health Organization on November 26, K417N was present in Omicron but absent in Delta, the predominant variant in Denmark at the time (*4*). K417N has been detected in 69.3% of Omicron overall but in 94.9% of the dominant sublinage in Denmark, BA.2 (*5*).

On November 30, samples from 3 WWTPs showed weak positive signals for the K417N mutation; cycle threshold values were 38.6 for WWTP1, 37.2 for WWTP2, and 39.9 for WWTP2. Cycle threshold values from the K417R assay were 32.1 for WWTP1, 32.5 for WWTP2, and 36.8 for WWTP2. A quantitative RT-PCR targeting the RNA dependent RNA polymerase gene determined viral loads for the 3 samples to be 5,400 genomes/L for WWTP1, 5,800 genomes/L for WWTP2, and 3,000 genomes/L for WWTP3. Only WWTP2 had suspected infection with Omicron among persons living in the catchment area (based on a spike gene dropout PCR performed at the Danish National COVID Test Center). For WWTP1 and WWTP3, the closest suspected case-patients resided 20 km from the catchment area.

Because Delta cases that have the K417N aa substitution have been detected sporadically in Denmark, in addition to the limitations mentioned above, the K417N variant PCR is not sufficient to confirm the presence of Omicron in wastewater samples. Therefore, purified RNA from K417N positive samples was transported to SSI by courier for confirmation by sequencing. A metagenomics approach was used, amplifying a 1,049-nt fragment of the spike gene, including part of the receptor-binding domain (nt 22799–23847 [GenBank accession no. NC_045512.2_Wuhan-Hu-1], aa 412‒761).

We used a modification of a protocol developed for Sanger sequencing (*6*). In brief, we used a Superscript IV One-Step PCR (Invitrogen, https://www.thermofisher.com). The PCR mixture contained 10 μL Platinum SuperFi RT-PCR Master Mix, 1 μL each of primers nCoV-2019_76_LEFT_alt3 and nCoV-2019_78_RIGHT (final concentration 0.4 µmol/L) artic primers v3 (*7*), 0.5 μL SuperScript IV RT Mix, 1.5 μL nuclease-free water, and 5 μL 5× diluted RNA from wastewater samples. PCR conditions were as reported (*5*). PCR products were bead purified before library preparation using Agencourt AMPure XP (Beckman Coulter, https://www.beckmancoulter.com).

We prepared libraries by using the Rapid Barcoding Sequencing Kit (Oxford Nanopore Technologies, https://nanoporetech.com) according to the manufacturer’s protocol and omitting optional steps. Libraries were loaded onto R9.4.1 flow cells (Oxford Nanopore Technologies). We performed sequencing on a GridION (Oxford Nanopore Technologies) by using high-accuracy basecalling. We analyzed generated reads continuously every hour for the first 3 hours and mapped reads against references, including the Delta and Omicron variants. We performed mapping and consensus extractions by using CLC Genomics Workbench version 21.0.4 (Long Read Support [β] plugin; QIAGEN, https://www.qiagen.com). We used NextClade (*8*) for typing consensus sequences and mutation detection (Table).

**Table:** Results of read mapping and consensus sequence analysis for detection of SARS-CoV-2 Omicron B.1.1.529 variant in 2 wastewater treatment plants, Denmark*

Sequencing Time, hours	Wastewater treatment plant 1				Wastewater treatment plant 2		
	Mapped reads		Omicron mapping consensus mutations		Mapped reads		Omicron mapping consensus mutations
Delta	Omicron	Delta	Omicron
1	17122	783	T23104A, **C23202A, C23525T, C23604A**		21743	1292	**C23202A, C23525T, C23604A**
2	30980	1446	**T23075C, C23202A, C23525T, C23604A**		40040	2326	C22971T, **T23075C, C23202A, C23525T, C23604A**
3	41320	1967	**T23075C, C23202A, C23525T, C23604A**		58254	3453	G22992C, **C22995A, T23075C, C23202A, C23525T, C23604A**

*Boldface indicates Omicron-specific mutations. Mutation A23403G was omitted because it is present in Delta and Omicron, but it was present in all consensus sequences.

At every analysis point, 4.5% of reads mapped as Omicron at WWTP1 and 5.6% at WWTP2. Few (<100) reads from WWTP3 mapped to any SARS-CoV-2 references, probably because of low viral load. Within 1 hour of sequencing (≈3 hours after RNA samples arrived at SSI and 9 hours after wastewater sample collection), 3 identical Omicron-specific mutations (C23202A, C23525T, and C23604A) were detected in the consensus sequences of WWTP1 and WWTP2 (Table). After 2 hours, a single additional Omicron mutation was found in both WWTPs. For WWTP2, one additional mutation was detected after 3 hours (Table). Omicron confirmed by whole-genome sequencing was detected in humans in the catchment areas of WWTP1 on December 12 and WWTP2 December 6. Our results show that this rapid metagenomics-like method can detect SARS-CoV-2 variants n a small fraction of the population, even at low viral loads.

In conclusion, we have demonstrated wastewater surveillance for SARS-CoV-2 variants by using a setup combining mutation-specific, real-time PCR and rapid nanopore sequencing. This surveillance might serve as an early warning system in a scenario in which a known variant is emerging, even in areas with low incidence.
